# Molecular profiling of T-helper immune genes during dengue virus infection

**DOI:** 10.1186/1743-422X-5-165

**Published:** 2008-12-31

**Authors:** Jincheng Chen, Mary Mah Lee Ng, Justin Jang Hann Chu

**Affiliations:** 1 Department of Microbiology, Yong Loo Lin School of Medicine, 5 Science Drive 2, National University of Singapore, 117597, Singapore

## Abstract

In this study, we provide a comprehensive molecular profiling of the involvement of T- helper (Th) genes during dengue virus infection of different cell types. The Th gene profiles of three human cell types (monocytes, T-cells and hepatocytes) were analyzed simultaneously via array-based RT-PCR upon infection with dengue virus. Differential regulation of 41 Th genes was identified and of which 20 of those genes may contribute to immuno-pathogenesis of dengue virus infection by regulating inflammation, thrombocytopenia and vascular permeability. Among the strongly up-regulated genes were the RANTES, CC-CKR3, IRF4, CLEC2C, IL-6 and TLR6, which are potent inducer of inflammation and vascular permeability. Profiling genes obtained from this study may serve as potential biomarkers and the modulation of Th immune responses during dengue virus infection has important implications in disease outcome.

## Findings

Dengue virus (DENV) is a member of the *Flavivirus *genus of the *Flaviviridae *family of enveloped, positive-strand RNA viruses. Four distinct serotypes (DENV1-4) of dengue viruses are transmitted to humans through the bites of the mosquito species, *Aedes aegypti *and *A. albopictus*. DENV causes a spectrum of disease in human, from acute febrile illness dengue fever (DF) to life-threatening dengue hemorrhagic fever/dengue shock syndrome (DHF/DSS). It has been estimated that about 50–100 million cases of DF, and about 250,000–500,000 cases of DHF occur worldwide every year. Furthermore, 2.5 billion of people are at risk for infection in subtropical and tropical regions of the world in the absence of effective intervention [1,2]. It is hypothesized that immunological mechanisms play a key role in the pathogenesis of dengue infection. Although several gene expression profiling studies of dengue virus-infected cells have been carried out previously [3-6], little is currently known about the molecular mechanism of how Th genes and Th-related genes are activated and implicated in the immuno-pathogenesis of DENV infection.

In this study, we conducted a novel focused gene set, array-based platform of transcriptional Real Time (RT)-PCR analysis of human cell response to DENV infection during peak virus production and focused on genes which are related to the three classes of helper T cells. The three classes of 84 helper T cells (Th1-Th2-Th3) genes were simultaneously profiled using the RT-PCR array (Table 1). Comparative profiling of the Th genes was carried out on three different human cell types (monocytes, T-cells and hepatocytes). The data generated from the RT-PCR array profiling will enable us to determine the crucial virus-cell interactions that take place during DENV infection. The RT-PCR arrays revealed differential regulation of 41 Th genes among the different human cell types, these genes include cytokine genes representative of Th1, Th2 and Th3 cells, transcriptional factors that regulate the expression of these cytokines as well as other markers of CD4+ T lymphocytes. Genes involved in immune cell activation and the Th1 and Th2 type immune responses were also noted. From these, 20 genes were identified and these genes may contribute to the immuno-regulation of DENV pathogenesis. This study provided a first insight to the differential regulation of Th genes during DENV infection of different human cell types.

**Table 1:** Complete list of genes tested in the Th genes RT-PCR array.

No.	Gene
1	B-cell CLL/lymphoma 3
2	Chemokine (C-C motif) ligand 11
3	Chemokine (C-C motif) ligand 5
4	Chemokine (C-C motif) ligand 7
5	Chemokine (C-C motif) receptor 2
6	Chemokine (C-C motif) receptor 3
7	Chemokine (C-C motif) receptor 4
8	Chemokine (C-C motif) receptor 5
9	CD28 molecule
10	CD4 molecule
11	CD40 ligand (TNF superfamily, member 5, hyper-IgM syndrome)
12	CD69 molecule
13	CD80 molecule
14	CD86 molecule
15	CCAAT/enhancer binding protein (C/EBP), beta
16	CREB binding protein (Rubinstein-Taybi syndrome)
17	Colony stimulating factor 2 (granulocyte-macrophage)
18	Cytotoxic T-lymphocyte-associated protein 4
19	Chemokine (C-X-C motif) receptor 3
20	Fas ligand (TNF superfamily, member 6)
21	GATA binding protein 3
22	Growth factor independent 1
23	Glomulin, FKBP associated protein
24	G protein-coupled receptor 44
25	Hepatitis A virus cellular receptor 2
26	Inducible T-cell co-stimulator
27	Interferon, gamma
28	Immunoglobulin superfamily, member 6
29	Interleukin 10
30	Interleukin 12B
31	Interleukin 12 receptor, beta 2
32	Interleukin 13
33	Interleukin 13 receptor, alpha 1
34	Interleukin 15
35	Interleukin 18 (interferon-gamma-inducing factor)
36	Interleukin 18 receptor 1
37	Interleukin 1 receptor, type I
38	Interleukin 1 receptor, type II
39	Interleukin 2
40	Interleukin 2 receptor, alpha
41	Interleukin 4
42	Interleukin 4 receptor
43	Interleukin 5 (colony-stimulating factor, eosinophil)
44	Interleukin 6 (interferon, beta 2)
45	Interleukin 6 receptor
46	Interleukin 7
47	Interleukin 9
48	Inhibin, alpha
49	Inhibin, beta A (activin A, activin AB alpha polypeptide)
50	Interferon regulatory factor 1
51	Interferon regulatory factor 4
52	Janus kinase 1 (a protein tyrosine kinase)
53	Janus kinase 2 (a protein tyrosine kinase)
54	Lymphocyte-activation gene 3
55	Linker for activation of T cells
56	V-maf musculoaponeurotic fibrosarcoma oncogene homolog (avian)
57	Mitogen-activated protein kinase kinase 7
58	Mitogen-activated protein kinase 8
59	Nuclear factor of activated T-cells, cytoplasmic, calcineurin-dependent 1
60	Nuclear factor of activated T-cells, cytoplasmic, calcineurin-dependent 2
61	Nuclear factor of activated T-cells, cytoplasmic, calcineurin-dependent 2 interacting protein
62	Polycomb group ring finger 2
63	Protein tyrosine phosphatase, receptor type, C
64	Surfactant, pulmonary-associated protein D
65	Suppressor of cytokine signaling 1
66	Suppressor of cytokine signaling 2
67	Suppressor of cytokine signaling 5
68	Secreted phosphoprotein 1 (osteopontin, bone sialoprotein I, early T-lymphocyte activation 1)
69	Signal transducer and activator of transcription 1, 91kDa
70	Signal transducer and activator of transcription 4
71	Signal transducer and activator of transcription 6, interleukin-4 induced
72	T-box 21
73	Transcription factor CP2
74	Transforming growth factor, beta 3
75	Toll-like receptor 4
76	Toll-like receptor 6
77	Transmembrane emp24 protein transport domain containing 1
78	Tumor necrosis factor (TNF superfamily, member 2)
79	CD27 molecule
80	Tumor necrosis factor receptor superfamily, member 8
81	Tumor necrosis factor receptor superfamily, member 9
82	Tumor necrosis factor (ligand) superfamily, member 4
83	Tyrosine kinase 2
84	YY1 transcription factor

**Figure 1:**
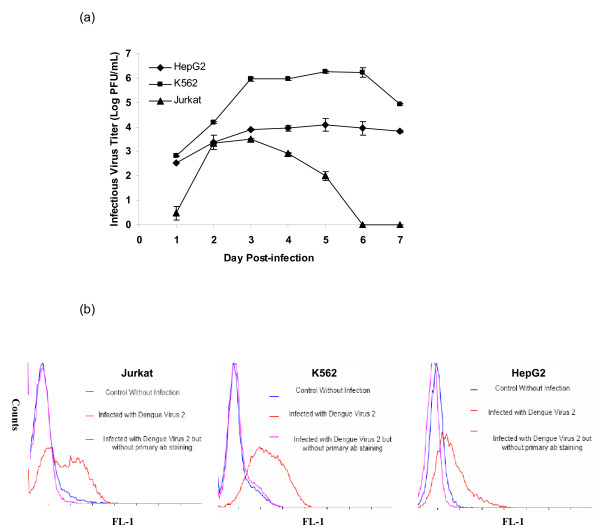
**Infectivity of human cell lines with DENV**. (a) Growth curves of DENV in different human cell types. (b) Detection of DENV viral antigen (envelope protein) in different human cell types via flow cytometry analysis. All the human cells were shown to support DENV replication and high infectious virus titers were also obtained from the cells at 3 days post-infection.

Dengue virus is known to be able to infect different kinds of cell types in human [7]. In this study, the susceptibility of human cell lines, K562, Jurkat and HepG2 to DENV 2 virus infection was first determined. K562 is a myelogenous cell line with monocyte and granulocyte properties [8]. Jurkat is a T cell lymphoblast-like cell line [9] and HepG2 is a heptocellular liver cell line [10]. Both K562 and Jurkat cells were selected for analysis as they are of immune origin and the HepG2 cell line was selected since dengue virus is hepatotrophic. The use of human cell lines for this study has been given approval by the National University of Singapore Institutional Review Board. These human cell lines were first subjected to low passage human isolate DENV (serotype 2, Singapore strain Den2ST) infection at a multiplicity of infection of 10. At different time points post-infection, virus containing supernatant were harvested for plaque assays and the DENV-infected cells were stained for immunofluorescence detection of viral antigen (envelope protein) production via flow cytometry. All the three human cell lines are shown to be highly susceptible to dengue virus infection, producing reasonably high virus titers by 3 days post-infection (Figure 1a). Production of viral antigen was also detected in all the three cell lines by 3 days post-infection (Figure 1b).

Next, all the three human cell lines were infected with DENV2 and processed for RT-PCR array experiments. Three independent DENV infection experiments were carried out to ensure the reproducibility of the changes in gene expression. Total RNA of the human cells (K562/Jurkat/HepG2) was extracted using the Qiagen RNeasy Kit. The quality and integrity of the total RNA extracted from mock-infected and dengue virus-infected cells on day 3 post-infection was first checked by running a portion of the total RNA on 1% agarose gel to detect for human ribosomal RNA, 28S and 18S. Human Th1-Th2-Th3 RT^2 ^Profiler™ PCR Array (Superarray) was used for the simultaneous profiling of 84 genes related to the three classes of helper T cells. RT^2 ^First Strand Kit (Superarray) was used for reverse transcription to obtain cDNA from total RNA. Five housekeeping genes (β-2-microglobulin, hypoxanthine phosphoribosyltransferase 1, ribosomal protein L13a, glyceraldehyde-3-phosphate dehydrogenase and β-actin) were included in the array to minimize functional biases.

**Figure 2:**
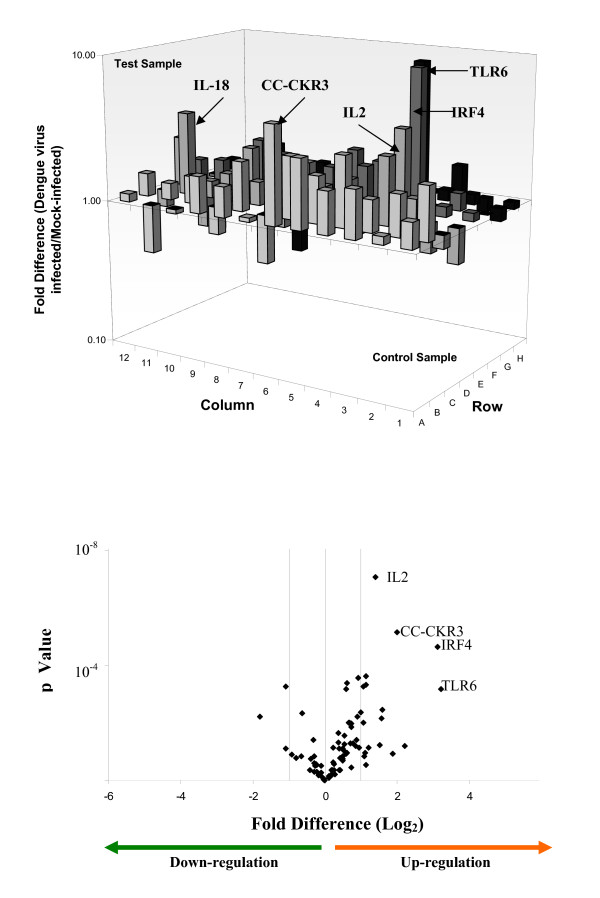
**Expression and statistical validation profiles of Th genes in dengue virus infected Jurkat cells**. The differential regulation (up or down-regulated) of the 84 Th and Th-related genes upon dengue virus infection of Jurkat cells are shown. Genes with greater than 3Log_2 _fold increase are indicated in the respective graphs of each cell types. The volcano plot of the RT-PCR array for each of the cell types is also provided. The plot arranges Th genes along dimensions of differential regulation (either up or down-regulation – X axis) and statistical significance (Y-axis). The higher values on the Y-axis indicate statistical significant of the up or down-regulated genes.

**Figure 3:**
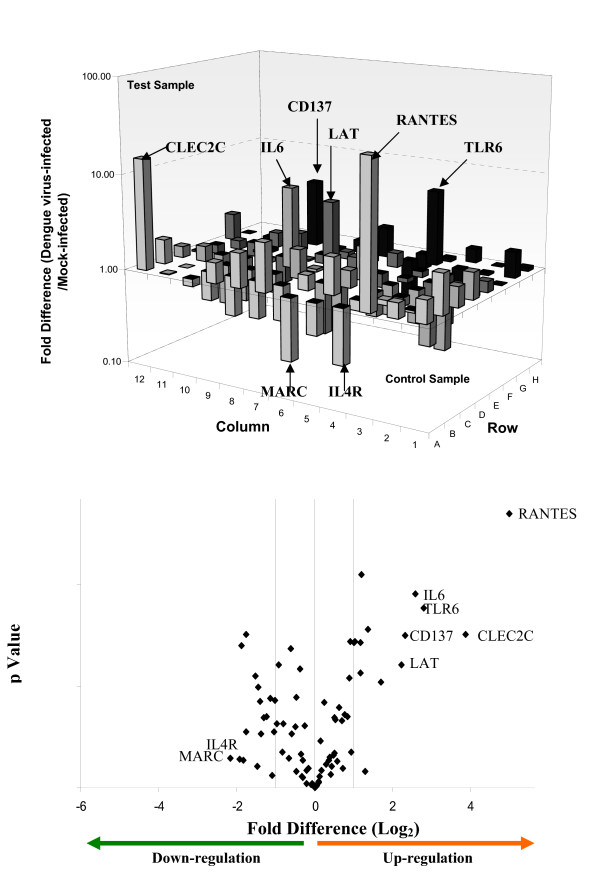
**Expression and statistical validation profiles of Th genes in dengue virus infected K562 cells**. The differential regulation (up or down-regulated) of the 84 Th and Th-related genes upon dengue virus infection of K562 cells are shown. Genes with greater than 3 Log_2_fold increase are indicated in the respective graphs of each cell types. The volcano plot of the RT-PCR array for each of the cell types is also provided. The plot arranges Th genes along dimensions of differential regulation (either up or down-regulation – X axis) and statistical significance (Y-axis). The higher values on the Y-axis indicate statistical significant of the up or down-regulated genes.

**Figure 4:**
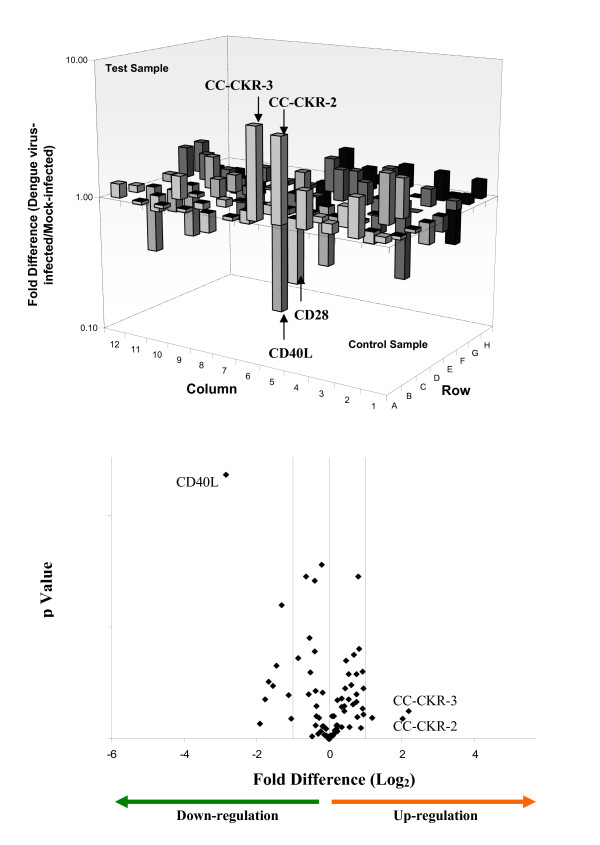
**Expression and statistical validation profiles of Th genes in dengue virus infected Hep G2 cells**. The differential regulation (up or down-regulated) of the 84 Th and Th-related genes upon dengue virus infection of HepG2 cells are shown. Genes with greater than 3 Log_2_fold increase are indicated in the respective graphs of each cell types. The volcano plot of the RT-PCR array for each of the cell types is also provided. The plot arranges Th genes along dimensions of differential regulation (either up or down-regulation – X axis) and statistical significance (Y-axis). The higher values on the Y-axis indicate statistical significant of the up or down-regulated genes.

Genes with fold value more than 2 in absolute value were considered to be differentially regulated. P-value of 3 independent control experiments and test experiments obtained for each gene was calculated. The differential regulation of the Th genes upon DENV infection of the human cell types are tabulated in Tables 2, 3 and 4. The Th genes are further classified based on their potential pathogenesis-induced classes (immune cell activation, inflammation, thrombocytopenia and vascular permeability) with reference to the description of the gene function summarized from Online Mendelian Inheritance in Man (OMIM), National Center for Biotechnology Information, National Library of Medicine  as of November 2008. From these genes, a group of 20 genes that may provide the molecular basis of the observed pathogenesis in the human cells upon DENV infection was identified (Tables 2, 3 and 4 – bolded). The criterion for the selection of these genes was based on their ability to enhance immune cell activation, inflammatory responses, thrombocytopenia as well as vascular permeability upon differential regulation. The complete gene expression profiles of all the three cell lines infected with DENV were provided (Figures 2, 3 and 4). The Th genes that were either up or down-regulated significantly in the different cell types were also indicated in the volcano plots as shown in Figures 2, 3 and 4. RANTES, CC-CKR-3, TLR6 and IL-6 were highly up-regulated in Jurkat and K562 cells upon infection with dengue virus. CD40L was shown to be significantly down-regulated in both Jurkat and HepG2 cells upon dengue virus infection.

**Table 2:** Differentially regulated Th genes of Jurkat cells infected with DENV.

Gene	Gene name	Fold Change	Immune cell activation	Inflammatory responses	Thrombo-cytopenia	Vascular permeability
Th1 immune response						
**CSF2**	**GMCSF**	**2.08**	**✔**	**✔**	**✔**	**✔**
**APT1LG1/CD178**	**FASLG**	**2.18**		**✔**		
INHA	Inhibin, alpha	2.19	✓			
**CLMF/CLMF2**	**Interleukin 12B**	**2.20**	**✔**	**✔**		
CD80	CD28LG/CD28LG1	2.29	✓			
**IFNG**	**Interferon, gamma**	**2.85**		**✔**	**✔**	**✔**
**IL18**	**Interleukin 18**	**3.69**	**✔**	**✔**		**✔**
**IL2**	**Interleukin 2**	**3.97**	**✔**	**✔**	**✔**	**✔**
**TLR6**	**Toll-like receptor 6**	**8.68**	**✔**	**✔**	**✔**	**✔**
CD182/CD183	CXCR3	-2.12	✓	✓		
Th2 immune response						
**RANTES**	**Chemokine ligand 5**	**2.11**	**✔**	**✔**	**✔**	**✔**
**CC-CKR-2/CCR2A**	**Chemokine receptor 2**	**2.95**		**✔**	**✔**	**✔**
**CBP/RSTS**	**CREB binding protein**	**3.00**		**✔**		
**CC-CKR-3/CD193**	**Chemokine receptor 3**	**4.64**		**✔**		**✔**
Th1\Th2 immune response						
**BSF2/HGF**	**Interleukin 6**	**2.16**	**✔**	**✔**	**✔**	**✔**
**CTLA4**	**Cytotoxic T-lymphocyte-associated protein 4**	**2.64**		**✔**		
**IRF4**	**Interferon regulatory factor 4**	**9.27**	**✔**	**✔**		
CD154/CD40L	CD40 ligand	-2.13	✓	✓	✓	✓
**4-1BB/CD137**	**Tumor necrosis factor receptor superfamily**	**-3.49**	**✔**	**✔**		**✔**
Transcriptional Factors						
**SOCS2**	**Suppressor of cytokine signaling 2**	**2.09**		**✔**	**✔**	

**Table 3:** Differentially regulated Th genes of K562 cells infected with DENV.

Gene	Gene name	Fold Change	Immune cell activation	Inflammatory responses	Thrombo-cytopenia	Vascular permeability
Th1 immune response						
RP11-102M16.1	Interleukin 12 receptor	2.01	✓		✓	
**DIF/TNF-alpha**	**Tumor necrosis factor α **	**2.06**		**✔**		**✔**
**CC-CKR-5/CCCKR5**	**Chemokine receptor 5**	**2.30**		**✔**		**✔**
**CSF2**	**GMCSF**	**2.45**	**✔**	**✔**	**✔**	**✔**
CD80	CD28LG/CD28LG1	2.60	✓			
**TLR6**	**Toll-like receptor 6**	**6.04**	**✔**	**✔**	**✔**	**✔**
**CD69**	**CLEC2C**	**14.62**	**✔**	**✔**	**✔**	**✔**
**IL2**	**Interleukin 2**	**-2.20**	**✔**	**✔**	**✔**	**✔**
APT1LG1/CD178	FASLG	-2.48		✓		
CLMF/CLMF2	Interleukin 12B	-3.35	✓	✓		
CD121b/IL1RB	Interleukin 1 receptor	-3.67			✓	
Th2 immune response						
**CC-CKR-2/CCR2A**	**Chemokine receptor 2**	**2.14**		**✔**	**✔**	**✔**
**CC-CKR-4/CKR4**	**Chemokine receptor 4**	**3.25**	**✔**	**✔**	**✔**	**✔**
**CC-CKR-3/CD193**	**Chemokine receptor 3**	**4.44**		**✔**		**✔**
**RANTES**	**Chemokine ligand 5**	**31.78**	**✔**	**✔**	**✔**	**✔**
**BSF1/IL-4**	**Interleukin 4**	**-2.25**	**✔**	**✔**	**✔**	**✔**
HDR	GATA binding protein 3	-2.61	✓	✓		
IL1RL1LG/Il1rl1l	Transmembrane emp24 protein transport domain	-2.74			✓	
AILIM/CD278	Inducible T-cell co-stimulator	-2.75	✓			
CD124/IL4RA	Interleukin 4 receptor	-3.52	✓	✓		
FIC/MARC	Chemokine ligand 7	-3.76	✓	✓		✓
Th1\Th2 immune response						
LAT	Linker for activation of T cells	4.69	✓			
**4-1BB/CD137**	**Tumor necrosis factor receptor superfamily**	**4.98**	**✔**	**✔**		**✔**
**BSF2/HGF**	**Interleukin 6**	**6.96**	**✔**	**✔**	**✔**	**✔**
IL-7	Interleukin 7	-2.87	✓	✓	✓	
PTPRC	Protein tyrosine phosphatase, receptor type, C	-3.39	✓	✓	✓	
Transcriptional Factors						
**SOCS2**	**Suppressor of cytokine signaling 2**	**2.27**		**✔**	**✔**	
ZNF163	Growth factor independent 1	-2.01	✓	✓		

**Table 4:** Differentially regulated Th genes of HepG2 hepatocytes infected with DENV.

Gene	Gene name	Fold Change	Immune cell activation	Inflammatory responses	Thrombo-cytopenia	Vascular permeability
Th1 immune response						
S152/T14	CD 27	-2.06	✓			
CSF2	GMCSF	-2.91	✓	✓	✓	✓
CDw218a/IL-1Rrp	Interleukin 18 receptor 1	-3.15				
Tp44	CD28	-7.07	✓			
CD154/CD40L	CD40 ligand	-9.20	✓	✓	✓	✓
Th2 immune response						
AILIM/CD278	Inducible T-cell co-stimulator	2.28	✓			
**CC-CKR-2/CCR2A**	**Chemokine receptor 2**	**4.08**		**✔**	**✔**	**✔**
**CC-CKR-3/CD193**	**Chemokine receptor 3**	**4.54**		**✔**		**✔**
HP40/IL-9	Interleukin 9	-2.49	✓	✓		✓
BSF1/IL-4	Interleukin 4	-2.73	✓	✓	✓	✓
EDF/IL-5	Interleukin 5	-7.12	✓	✓		
Th1\Th2 immune response						
PTPRC	Protein tyrosine phosphatase, receptor type, C	-3.73	✓	✓	✓	
4-1BB/CD137	Tumor necrosis factor receptor superfamily	-3.41	✓	✓		✓
Transcriptional Factors						
STAT4	Signal transducer and activator of transcription 4	-2.17	✓	✓		

Dengue virus infection causes dengue fever (DF), dengue hemorrhagic fever (DHF), and dengue shock syndrome (DSS), whose pathogenesis are not clearly understood. Current hypotheses of antibody-dependent enhancement, virus virulence, and IFN-gamma/TNF α-mediated immuno-pathogenesis are insufficient to explain clinical manifestations of DHF/DSS such as thrombocytopenia and hemoconcentration [11]. Furthermore, Chaturvedi and co-workers (1999) documented a shift from a Th1 response in mild dengue to a Th2 response in severe dengue haemorrhagic fever [12]. Increased serum levels of IL-4, IL-6 and IL-10 were observed mainly in cases of DHF grades III and IV. In contrast, the levels of IFN-γ and IL-2 were highest in cases of DF and low in DHF grade IV. Hence, these data indicated the importance of Th genes in mediating the pathogenesis of DENV infection.

In this study, we have been able to identify 20 Th and Th-related genes from the RT-PCR arrays that may contribute to the molecular basis of DENV pathogenesis. The 20 genes were selected based on their differential regulation and their implications in mediating immune cell activation, inflammatory responses, thrombocytopenia and vascular permeability during DENV infection. Nevertheless, the involvement of these genes on the molecular pathogenesis of DENV infection warrant further cohort studies on patients with DENV infection and these studies are currently being conducted in our laboratory.

Jurkat and K562 cells being immune cells have more differentially regulated Th related genes upon DENV infection when compared with HepG2 hepatocytes (Tables 2, 3 and 4). Fas ligand, IL-2 and IFN-γ up-regulation in Jurkat cells were also reported in studies using DENV-infected T cell clones [13]. Up-regulation of RANTES and IL-6 in dengue 2 virus-infected K562 cells was also reported by King and co-workers, showing elevated RANTES and IL-6 in DENV-infected mast cells and monocytes [14]. An earlier study by Liu and co-worker reported the expression of CD69 on monocytes at day 4 after the onset of fever which also substantiated the observation of CD69 up-regulation in DENV-infected K562 cells in this study [15]. Hence, the consistency of the data obtained from this study with previous published studies provided this experimental approach with confidence.

The observation of the few similar Th related gene regulation between the three cell lines suggested that the DENV triggered Th gene expression that was cell-type dependent in the RT-PCR array. Another gene profiling study performed on DENV-infected cells also observed cell-type specific gene changes [6]. Nevertheless, a group of Th1 and Th2 genes inducing cell activation and inflammation (GMCSF, RANTES, TLR6 and CC-CKR-3) were found to be significantly up-regulated in both K562 and Jurkat cells, this may imply the presence of potential cross-talking pathways that mediate common inflammatory responses during DENV infection. More studies are now being conducted to further verify this interesting observation. The over-expression or inappropriate inflammation can lead to tissue destruction commonly observed in DSS/DHF [16].

For dengue virus infection, the severity of the disease is best indicated by the extent of plasma leakage. A major group of genes (GMCSF, RANTES, IL2, INFγ, TLR6, CCR2A, IL-6, CKR-4) causing thrombocytopenia and vascular permeability was also found to be up-regulated in all three cell types. These gene products are known mediators of thrombocytopenia and vascular permeability. In consistent with previous studies, some of these genes have also been reported to be up-regulated during dengue infection, including RANTES [17] and IL-6 [18]. In addition, the down-regulation of IL-4 was observed in K562 cells and this has important implication in causing vascular permeability [19]. The over-expression of these cellular Th genes might play a crucial role in enhanced production of anti-platelet or anti-endothelial cell autoantibodies, elevated levels of tPA, as well as a deficiency in coagulation. Therefore, damaged endothelial cells may upset the procoagulant/anticoagulant balance of endothelium and increase the tendency of bleeding. Activated endothelial cells may also contribute to the development of thrombocytopenia by sequestering platelets [20].

In summary, the focused Th genes RT-PCR array profiling showed a complex network of DENV-induced cell interactions during infection. The array-based Th gene profiles documented in this study may also serve as potential biomarkers for DENV infection. Nevertheless, further verification of these differentially regulated Th genes on DENV-infected patient samples will be helpful. Comparative profiling of Th genes in different human cell types in response to DENV infection may therefore provide interesting insights into the possible molecular mechanisms behind the observed cyto-pathology in DENV infection.

## Competing interests

The authors declare that they have no competing interests.

## Authors' contributions

JJHC designed research; JCC and JJHC performed research; JCC, JJHC and MLN analyzed data and wrote the paper.
